# Do it yourself: fabricating and evaluating a mosquito (Diptera: Culicidae) blood-feeding device to replace a commercial option

**DOI:** 10.1093/jisesa/iead072

**Published:** 2023-08-11

**Authors:** M Andrew Dewsnup, Ary Faraji, Gregory S White, Christopher S Bibbs

**Affiliations:** Salt Lake City Mosquito Abatement District, 2215 North 2200 West, Salt Lake City, UT 84116, USA; Salt Lake City Mosquito Abatement District, 2215 North 2200 West, Salt Lake City, UT 84116, USA; Salt Lake City Mosquito Abatement District, 2215 North 2200 West, Salt Lake City, UT 84116, USA; Salt Lake City Mosquito Abatement District, 2215 North 2200 West, Salt Lake City, UT 84116, USA

**Keywords:** artificial membrane feeder, rearing, blood feeding, vector, Hemotek

## Abstract

Tools for rearing hematophagous insects, such as mosquitoes (Diptera: Culicidae), in an insectary are essential for research and operational evaluations in vector biology and control. There is an abundance of low-cost options for practitioners without conventional infrastructure. However, few midrange options exist that provide a balance of efficiency and low material waste. We present here a reproducible design for an electrically powered blood-feeding device that offers long-term reusability, low material waste, and customizability for different species or experiments. The limitation is the requirement for electricity, but the gain is a simple, low-skill device that can be modified as needed. To validate the design, assessments of feeding angle and blood-feeding success were compared between the Salt Lake City Mosquito Abatement District artificial membrane feeder (SLAM) and a commercial system (Hemotek). Engorgement in *Aedes aegypti* (80–90%), *Culex pipiens* (50–80%), and *Culex tarsalis* (30–75%) was similar between the 2 units, resulting in nearly identical fecundity outcomes between devices. Additionally, 45° angles were more successful, generally, than presenting the feeders flat or vertical to the mosquitoes (df_3,48_, *P* = 1.014 × 10^−15^). This angle is simple to present with the SLAM device. Materials for in-house reproduction of the SLAM system are now widely available in regions with access to e-commerce and shipped goods. This results in a device schematic that should fit well into a relatively modular, do-it-yourself paradigm where the practitioner needs only to assemble some materials without complex engineering. This article provides schematics, cost comparison, and validation of the in-house-made SLAM system.

## Introduction

Research on hematophagous insects, such as mosquitoes (Diptera: Culicidae), is intrinsically limited by successful blood consumption during colony rearing or experiments. Historically, blood feeding of various insects can require host animals or volunteers offering an arm, which may also invite ethical scrutiny and complex regulations that may, or may not, directly influence the feasibility of the study (Achee et al. 2015). Aside from the logistical burden of acquiring exogenous blood meals, technical limitations on how to deliver the blood can also hamper scientific and operational investigations. In the case of mosquitoes, blood must be offered at or above body temperature and can compromise laboratory hygiene if not contained in a membrane or specialized device. The importance of accessible technology escalates when you consider that these devices can be an integral part of research beyond colony rearing, such as efficacy bioassays (Tsurukawa and Kawada 2014, Morimoto et al. 2021) or infectivity studies (Witmer et al. 2018, Sri-in et al. 2020). Improvements in technology have yielded designs for both disposable and reusable self-manufactured devices.

Most low-resource designs will repurpose disposable materials that are readily available (Tseng 2003, Costa-da-Silva et al. 2013, Finlayson et al. 2015, Siria et al. 2018, Chalida et al. 2020, Tyler-Julian et al. 2021, Faber et al. 2022). Unfortunately, they also generate waste from disposable material accumulation and often exhibit reduced integrity because of plasticware or other materials that age poorly. They can also be time consuming to construct enough of them to use routinely with large numbers of colonies. Single-use materials are advantageous when the alternative is unsuccessful blood feeding; for example, in countries where infrastructure or tools are limited (Costa-da-Silva et al. 2013, Finlayson et al. 2015, Siria et al. 2018, Chalida et al. 2020, Faber et al. 2022). However, there is a distinct absence of midrange devices that are scalable while also having a good balance of efficiency, ease of use, and low waste.

The most widely used midrange systems use recirculating water baths for balanced heating and modularization (Luo 2014, Dias et al. 2018, 2021, Faber et al. 2022). They also tend to require additional components, such as miscellaneous glassware, heating elements or hot plates, and tubing. There have been notable improvements in this with 3D-printed pieces that reduce the need for extra parts (Witmer et al. 2018). Despite that, the utility of water bath systems is often skill-gated, which reduces the success and efficiency by untrained staff. Even when practised in the operation, sometimes complex systems and those containing many moving parts are simply a nuisance for the practitioners, even if they get the job done. Furthermore, scalability is awkward with recirculating water systems, such as having many in-line duplicates (Dias et al. 2021, Faber et al. 2022), purposefully engineered glassware (Luo 2014), or cumbersome hands-on management requirements (Dhar et al. 2019, Tyler-Julian 2021). Water bath systems may also restrict reach across a room of cages while attached to the water tank.

Notably, fewer designs for electrically powered feeding units are available compared with some other methods of blood feeding (Benzon and Apperson 1987, Deng et al. 2012, Yatim et al. 2021). Unfortunately, the engineering required in electric systems may result in poor cost-effectiveness, particularly when operating under tight budgetary constraints. The main commercial option is the Hemotek membrane feeding system (Hemotek Ltd., Blackburn, UK), but few alternative versions are available and all are expensive (US$800–$2,000) with limited availability. Furthermore, scalability of those units is locked behind the purchase of proprietary subunits. In general, electrically powered feeders have long-term reusability, reducing disposal of single-use materials and possibly allowing reduced blood waste by tailoring reservoir sizes to not overuse blood-stock. In addition, it typically does not require oversight to ensure part integrity or manual rewarming by the operator. But the current reliance on the commercial model, Hemotek, creates barriers to entry from startup costs.

Using the United States as an example, most mosquito research operations have access to electricity. When the infrastructure is available, such programs can use active heating methods to reduce waste and complexity when working with blood for mosquito colony maintenance. But the distinct lack of affordable technology is an issue that strongly curbs the desirability of electric units. We present here the assembly of a low-range to midrange electric blood-feeder. The design is a composite of materials readily available through e-commerce and was validated on in-house colonies of mosquitoes at the Salt Lake City Mosquito Abatement District (SLCMAD).

## Materials and Methods

### Design and Construction

**Fig. 1.**
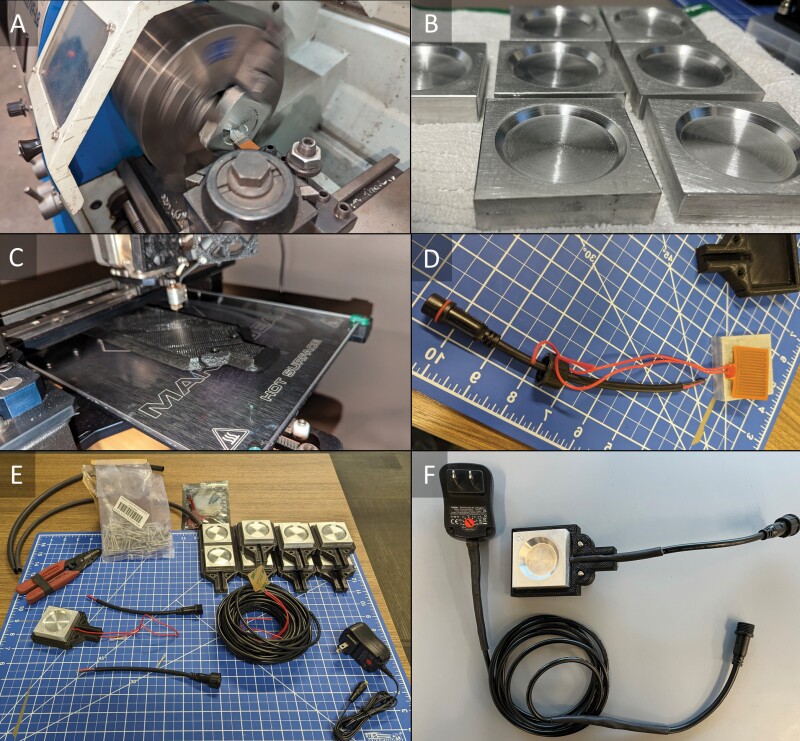
A) 5 × 5 × 1.3 cm solid aluminum square routed for a 3.8-cm-diameter well; B) aluminum wells with 0.16–0.32 cm depths, depending on needs; C) 3D-printed housing for mounting heater, wiring, and aluminum can be substituted for any cut housing with metal or plastic; D) 6-volt film heater with adhesive pad to adjoin the aluminum and quick connect wire harness to bridge to adapter; E) partial assembly of all components, showing option to extend wire length based on needs by braiding in additional equivalent gauge wire; F) final assembly.

A 5 cm wide by 1.3 cm high (2 in × 0.5 in) slab of 6061 aluminum was cut into 5 × 5 × 1.3 cm squares using a band saw. Cut squares were machine-routed to add wells of 0.16 cm depth for the purposes of study (Fig. 1). However, any depth, given the limitations of the metal, is feasible based on needs. A housing was 3D printed using PLA filament with 0.6-mm nozzle with set temperatures at 210 °C (410 °F) and 58 °C (136 °F) bed temperature. The STL (Standard Tessellation Language or Standard Triangle Language) file for the housing is uploaded to ThingiVerse under SLCMAD (https://www.thingiverse.com/slcmad/designs). In the absence of a 3D-printed housing, any cut housing of plastic or metal in a similar dimension to the STL file should suffice. A flat film adhesive heater (5-volt 1 watt, ICStation, Inc., Shenzen, China) was added to the backside of the aluminum squares. Screw-nut style, 2-pin, 18 AWG quick connect cables (IP65 Male/Female Connector, BTF-Lightning Technology Co, Ltd., Shenzhen, China) were spliced with the heater film wires and protected with water-resistant heat shrink (Dual Wall Marine Heat Shrink, Simple Electric Solutions LLC, Naples, FL). Heat shrink protected wire was slotted into the neck of the housing (Fig. 1), and the aluminum square was fitted into the receptacle. Clear-weld resin epoxy (J-B Weld Co, Marietta, GA) was then added to seal joints and gaps around the aluminum to prevent water intrusion. Additional 18 AWG wire can be spliced into the quick connect to lengthen the reach of the unit, again secured with water-resistant heat shrink, and then terminated after a final splice into an adjustable voltage universal AC/DC adapter (12 watt 4.5–12 volts, Belker Technology Co, Ltd., Newport Beach, CA).

**Fig. 2.**
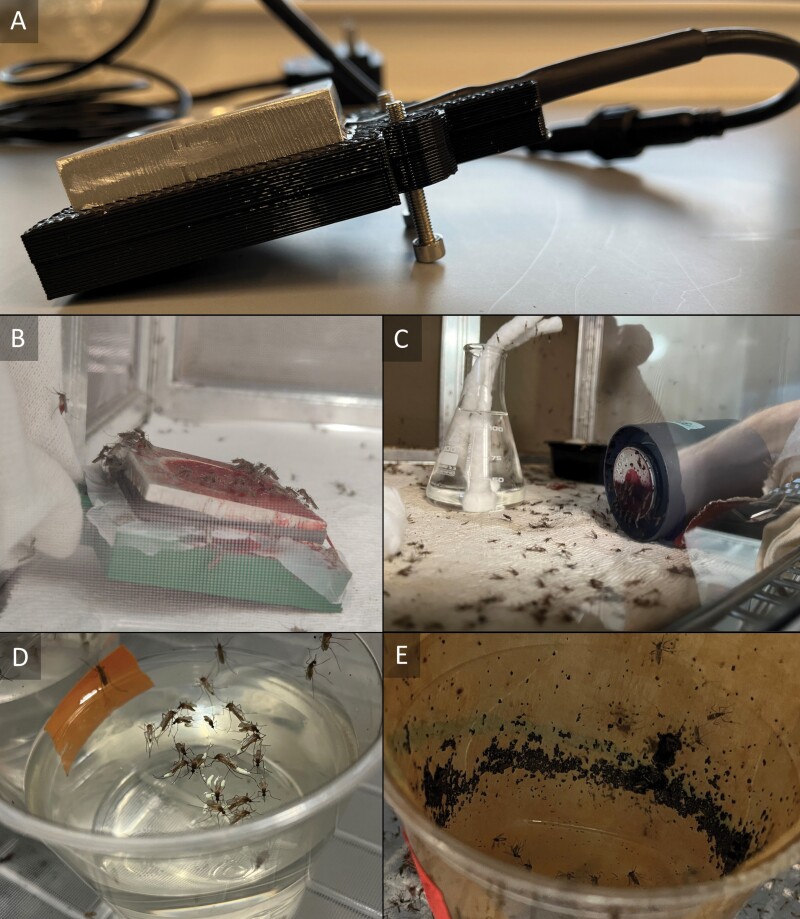
(A) Angle screws in the SLAMto allow height adjustment; B) MAD blood-feeder presented at an angle; C) Hemotek blood-feeder presented vertically; D) *Culex pipiens* egg raft assessment; E) *Aedes aegypti* egg paper assessment.

**Fig. 3.**
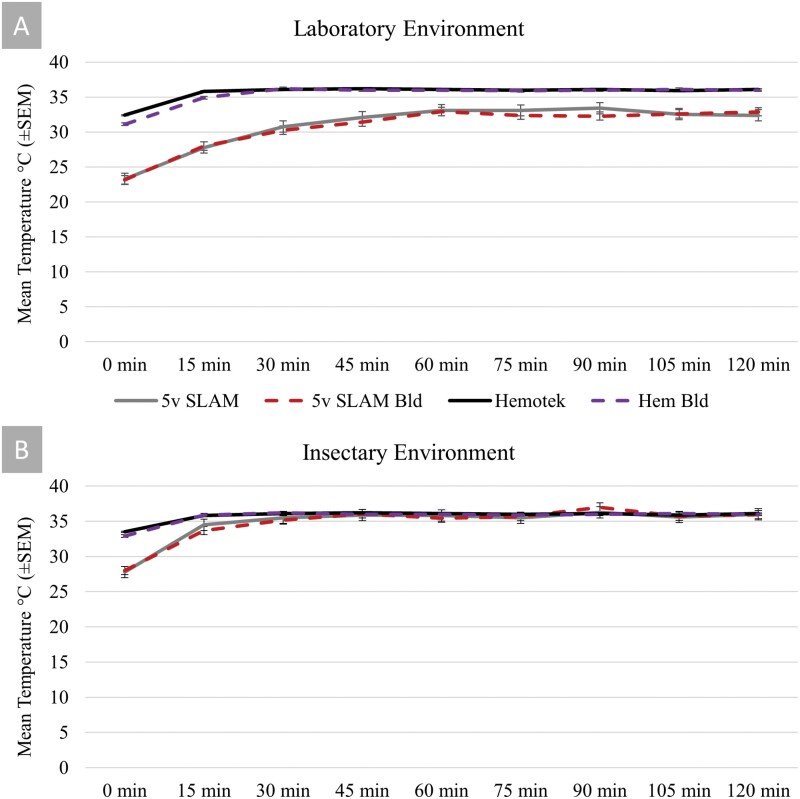
Temperature readings on the SLAM with a 5-volt setting and Hemotek system. Both devices were measured with or without blood. Data were taken in A) cool laboratory environment (22 ± 2 °C) and B) mosquito insectary (28 ± 2 °C). I-bars represent standard error of the mean.

When prepared with blood, a 5 cm (2 in) square of membrane (Parafilm M, Bemis Company, Inc., Neenah, WI) can be used to cover the filled well and secured with a rubber band (Fig. 2). The adjustable voltage dial on the AC adapter is the only form of temperature control for the house-made unit. At standard room temperature, ~22 ± 2 °C (70–75 °F), a 6-volt setting will yield blood warmed to ~35 ± 1 °C (94–97 °F). In a warmed room, such as a mosquito insectary held at 28 ± 2 °C (79–86 °F) air temperature, the house-made unit can be dropped to a 5-volt setting to maintain the same ~35 ± 1 °C (94–97 °F) blood temperature. Temperature settings were verified with a multimeter fitted with a temperature probe (MM600, Klein Tools, Inc., Lincolnshire, IL). Thermal stability using the 5-volt setting was verified at 15-min increments both with and without blood in laboratory (Fig. 3A) or insectary conditions (Fig. 3B). To adjust angle, the housing contained ports for knurled nuts (M4 × 8 × 6 Brass heat embedment nut, Hilitchi Co, Ltd., Shenzhen, China) that were slotted and then melted into place with a soldering iron. Then M4 size screws could be threaded at the neck of the wiring to allow manual adjustment of height (Fig. 2). Overall reach of the unit was 2 m (6 ft) from the plug, but can be made to any length based on how much wire is added to the assembly. The rough cost at the time of writing was ~$30 per unit.

### Colony Validation

All mosquitoes were reared and maintained in the SLCMAD insectaries. Species used for validation were 1952 Orlando strain *Aedes aegypti* (L.), 2016 SLCMAD strain *Culex pipiens* L., and the SAC-YOLO strain *Culex tarsalis* Coquillett (NR-43026, BEI Resources, NIAID, NIH). Mosquito larvae were reared in collection trays, and adult flight cages kept at consistent environmental conditions of 28 ± 1 °C temperature and 70 ± 5% relative humidity. Colony adults were fed on 10% sucrose solution ad libitum. Adults were allowed 5–10 days for mating before being offered their first blood-meal. All feeding assays were conducted in the aforementioned rearing conditions.

Cages of ~50 female and 50 male mosquitoes were offered sodium citrate treated whole bovine blood (Lampire Biological Laboratories, Inc., Pipersville, PA) in either a 3-ml Hemotek reservoir (Hemotek Ltd., Blackburn, UK) calibrated to 36 °C or a Salt Lake City Mosquito Abatement District artificial membrane feeder (SLAM) unit set to 5 volts. Verification with the multimeter probes validated the Hemotek to heat to 36.4 °C and the SLAM unit to heat to 35.8 °C. Both units were used with membranes (Parafilm M, Bemis Company, Inc., Neenah, WI) that were rubbed on arm and hand skin of a technician before stretching over the reservoirs and being perforated numerously using a minuten pin. Membranes were otherwise not preheated before use, and all feeding units were allowed 15 min of prewarming before being admitted to cages.

Feeding units were available for 30 min at either 0°, 45°, 90°, and 180° (resting on top of the cage) angles before removal. A tally of visibly engorged females was taken to measure feeding success. After 48 h for digestion and egg development, appropriate oviposition substrates were admitted to blood-fed cages. Cages were then allowed another 48 h to oviposit before substrates were tallied for general fecundity comparison (Fig. 2). This process was repeated with 3 separate cohorts of mosquitoes per species, per angle, and averaged for comparison between Hemotek and the SLAM units.

### Data Analysis

Data were analyzed using R statistical software (v.4.2.1, The R Foundation for Statistical Computing, Vienna, Austria) via RStudio (v. 3.3.0, RStudio PBC, Boston, MA). The loaded packages tidyverse, ggplot2, lme4, and emmeans were used to perform generalized linear models (GLM). Factorial combinations of species tested, feeding unit offered, and angle presented were used as treatment factors in analysis for blood-fed versus nonblood-fed results. Blood-feeding data was run in a GLM coded for binomial distribution with dispersion assumed to be known. Additional odds ratios were used to discriminate between device type for the included species. For eggs, Hemotek results were used as the comparison against the SLAM prototype, with combinations of species tested and angle presented as the treatment factors. Replicate number was used as a random effect in outputs for both blood-feeding success and egg yield. Fecundity data were run in a GLM coded for quasibinomial distribution with dispersion estimated, due to the large variance in egg counts within test groups.

## Results

**Fig. 4.**
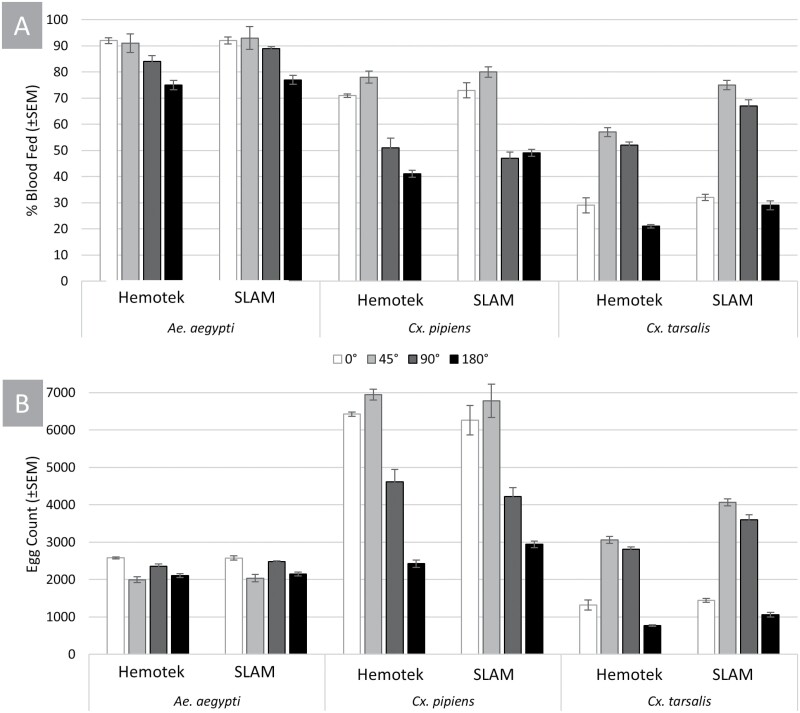
A) Blood-feeding response as mean % visibly engorged; B) Blood-feeding response as mean egg collection. I-bars represent standard error of the mean with data categorized based on species, then grouped for Hemotek blood-feeder and the SLAM.

Observationally, all mosquito species appeared immediately interested in the SLAM unit, with immediate landing and probing by all species. Comparatively, the Hemotek unit accumulated more mosquito activity as the 30-min interval progressed, while the SLAM unit was observed to be consistent over the same period. Blood-feeding success was similar between the Hemotek and the SLAM prototypes (Fig. 4A). There were no significant differences between devices when blood feeding at any angle with *Ae. aegypti* (*P* = 0.3624). Without considering device as a factor, assessments on angle of feeding alone indicated that blood-feeding success in *Ae. aegypti* was overall greatest at either 0° or 45° angles (df_3,48_, *P* = 1.014 × 10^−15^). In contrast, *Cx. pipiens* engorged to greater proportions on the SLAM units over the Hemotek, but only at the 180° (*P* = 3.15 × 10^−13^, fixed effect = 2.362, CI = ±1.333) and 45° angles (df_3,48_, *P* = 1.014 × 10^−15^). The largest changes were observed in *Cx. tarsalis*, with SLAM units yielding significantly greater blood feeding over the Hemotek for the 45° (*P* = 0.000193, fixed effect = 1.326, CI = ±1.764), 90° (*P* = 4.72 × 10^−7^, fixed effect = 1.749, CI = ±2.404), and 180° presentations (*P* = 2 × 10^−16^, fixed effect = 3.322, CI = ±1.764). Regardless of feeding unit with *Cx. tarsalis*, the 45° angle was most efficient (df_3,48_, *P* = 1.014 × 10^−15^). However, the overall trends and magnitudes were mirrored between both devices (Fig. 4A).

Trends within the egg count data deviate somewhat from the blood-feeding success (Fig. 4B). Despite decreasing receptivity to blood feeding at different angles from 0°, *Ae. aegypti* did not have a significant difference in fecundity between the 2 devices (*t* = 0.611, *P* = 0.547). When only evaluating angle, regardless of device, the feedings at 0° and 90° resulted in slight but significant differences in egg laying (Fig. 4B; *t* = 2.533, *P* = 0.0168) when compared with the 90° and 180° angles. When feeding *Cx. pipiens*, results between devices slightly favored the SLAM unit at 180° (*t* = 0.0361, fixed effect = −0.195, CI = ±0.213), but the actual egg yield was still less than what resulted from 0° or 45° angles, which were not significantly different between devices. *Culex tarsalis* fecundity mirrored the same trends as with blood-feeding success, with the greatest observed fecundity at 0° or 45° angles and a marginal bias to the SLAM unit overall at any of 45° (*t* = 0.0163, fixed effect = −0.286, CI = ±0.083), 90° (*t* = 0.0069, fixed effect = −0.249, CI = ±0.084), and 180° (*t* = 0.0163, fixed effect = −0.3194, CI = ±0.124) angles. Across the 3 species, fecundity was statistically higher in more comparisons with the SLAM than the Hemotek, albeit with quantitatively low differences in the egg numbers.

## Discussion

Using fecundity measures for *Ae. aegypti*, *Cx. pipiens*, and *Cx. tarsalis*, the experimental SLAM feeding unit was essentially equivalent to the Hemotek system. In the SLAM prototype, it is not ideal to adjust voltage for the temperature setting as opposed to dictating a specific value, as in the Hemotek system. Temperature precision was undoubtedly in favor of the Hemotek system because of a tuning/calibration option using their proprietary thermostat. However, it appeared through validation that the SLAM unit is sufficiently attractive and viable as long as the temperature management is not prohibitive. We acknowledge that there are cases where exact tuning may be mandatory, so as not to confound an experiment (Tsurukawa and Kawada 2014, Morimoto et al. 2021).

An overwhelming effort has been put into *Aedes* rearing studies using membrane feeders (Tseng 2003, Lyski et al. 2011, Deng et al. 2012, Costa-de-Silva et al. 2013, Luo et al. 2014, Finlayson et al. 2015, Siria et al. 2018, Almeida-Costa et al. 2020, Dias et al. 2021, Yatim et al. 2021, Faber et al. 2022), particularly with the encouragement of high-throughput rearing for sterile insect techniques (Gunathilaka et al. 2017). However, *Anopheles* species are included in comparatively fewer studies (Benzon and Apperson 1987, Tsurukawa and Kawada 2014, Finlayson et al. 2015, Siria et al. 2018, Dias et al. 2021). Interestingly, *Culex* spp. seem seldom included at all in artificial feeding studies (Dias et al. 2018, 2021, Tyler-Julian et al. 2021). Despite that *Culex* spp. are major arbovirus vectors, their lack of receptivity on artificial membranes (as little as half the comparative success) may be a reason for being less often included in membrane evaluations (Dias et al. 2021).

From our data, it did not appear that *Culex* spp. were specifically limited by either device, but it has also been found that angled surfaces and otherwise different orientations (Lyski et al. 2011) can influence feeding success with artificial membranes. That being the case, the Hemotek, with its cylindrical heater units (Fig. 2), can only easily be presented with a vertical surface (laying on the cage bottom) or top-down on the cage mesh. Additional angles can be managed by either hooking the power cable or nesting the device in a seat, such as a cardboard or plastic cup, but this is an unwanted step in the process even if it was performed for the sake of measurements in this study. The SLAM unit can be presented flat facing upwards, facing downwards on the cage mesh, or angled by shimming the unit with the attached screws. In all species tested, there appeared to be no downside to presenting at least a 45° angle for feeding. This was most noticeable in both *Culex* spp. and was much easier to accomplish with the SLAM device over the Hemotek. It should be noted, though, that the quirk of angle preference may be more relevant to field-collected mosquitoes, as colony mosquitoes can be forced to accept whatever methods are used in routine rearing.

Regardless of using the Hemotek or the SLAM unit, we believe that electric heaters confer the benefit of low user-error rates by having a more streamlined material commitment. In the SLAM unit’s case, the system is customizable for different species or experiments and allows for relatively cheap standardized operation. In addition, refinements in the model can be made with 3D-printed components, additional metal pieces, or prefabricated plastic housing. Given the benefits, there are also clear limitations that this method will not work for high-throughput rearing, such as in Gunathilaka et al. (2017) because of limited outlets and total available power within a rearing room. In such cases, the disposable or low-resource methods are liable to have the best logistics. For small- and mid-sized rearing efforts, such as those performed in most laboratories and operational abatement programs, we believe the SLAM feeding unit is a viable competitor to the Hemotek system and, just as with Hemotek, reduces waste by limiting single-use materials to just the membrane and blood parts. Perhaps more important, the SLAM unit could allow some mosquito species to be more accessible through its customization. Overall, we believe the design improves the accessibility of electric blood feeders to small organizations and may lead to higher availability and productivity to further enhance hematophagous insect investigations.
